# Dietary *Epimedium* extract supplementation improves intestinal functions and alters gut microbiota in broilers

**DOI:** 10.1186/s40104-022-00812-1

**Published:** 2023-01-18

**Authors:** Jiaqi Zhang, Haitao Yu, Huiyan Zhang, Qingyu Zhao, Wei Si, Yuchang Qin, Junmin Zhang

**Affiliations:** 1 State Key Laboratory of Animal Nutrition, Institute of Animal Sciences, Chinese Academy of Agricultural Sciences, 100193 Beijing, China; 2 Scientific Observing and Experiment Station of Animal Genetic Resources and Nutrition in North China of Ministry of Agriculture and Rural Affairs, Institute of Animal Sciences of Chinese Academy of Agricultural Sciences, 100193 Beijing, China

**Keywords:** Antioxidation, Broilers, *Epimedium*, Growth performance, Microbiota

## Abstract

**Background:**

Growth-promoting antibiotics have been banned by law in the livestock and poultry breeding industry in many countries. Various alternatives to antibiotics have been investigated for using in livestock. *Epimedium* (EM) is an herb rich in flavonoids that has many beneficial effects on animals. Therefore, this study was planned to explore the potential of EM as a new alternative antibiotic product in animal feed.

**Methods:**

A total of 720 1-day-old male broilers (Arbor Acres Plus) were randomly divided into six groups and fed basal diet (normal control; NC), basal diet supplemented with antibiotic (75 mg/kg chlortetracycline; CTC), and basal diet supplemented with 100, 200, 400 or 800 mg/kg EM extract for 6 weeks (EM100, EM200, EM400 and EM800 groups). The growth performance at weeks 3 and 6 was measured. Serum, intestinal tissue and feces were collected to assay for antioxidant indexes, intestinal permeability, lactic acid and short-chain fatty acids (SCFAs) profiles, microbial composition, and expression of intestinal barrier genes.

**Results:**

The average daily feed intake in CTC group at 1–21 d was significantly higher than that in the NC group, and had no statistical difference with EM groups. Compared with NC group, average daily gain in CTC and EM200 groups increased significantly at 1–21 and 1–42 d. Compared with NC group, EM200 and EM400 groups had significantly decreased levels of lipopolysaccharide and *D*-lactic acid in serum throughout the study. The concentrations of lactic acid, acetic acid, propionic acid, butyric acid and SCFAs in feces of birds fed 200 mg/kg EM diet were significantly higher than those fed chlortetracycline. The dietary supplementation of chlortetracycline and 200 mg/kg EM significantly increased ileal expression of *SOD1*, *Claudin-1* and *ZO-1* genes. Dietary supplemented with 200 mg/kg EM increased the relative abundances of *g_NK4A214_group* and *Lactobacillus* in the jejunal, while the relative abundances of *Microbacterium*, *Kitasatospora*, *Bacteroides* in the jejunal and *Gallibacterium* in the ileum decreased.

**Conclusion:**

Supplementation with 200 mg/kg EM extract improved the composition of intestinal microbiota by regulating the core bacterial genus *Lactobacillus*, and increased the concentration of beneficial metabolites lactic acid and SCFAs in the flora, thereby improving the antioxidant capacity and intestinal permeability, enhancing the function of tight junction proteins. These beneficial effects improved the growth performance of broilers.

**Graphical Abstract:**

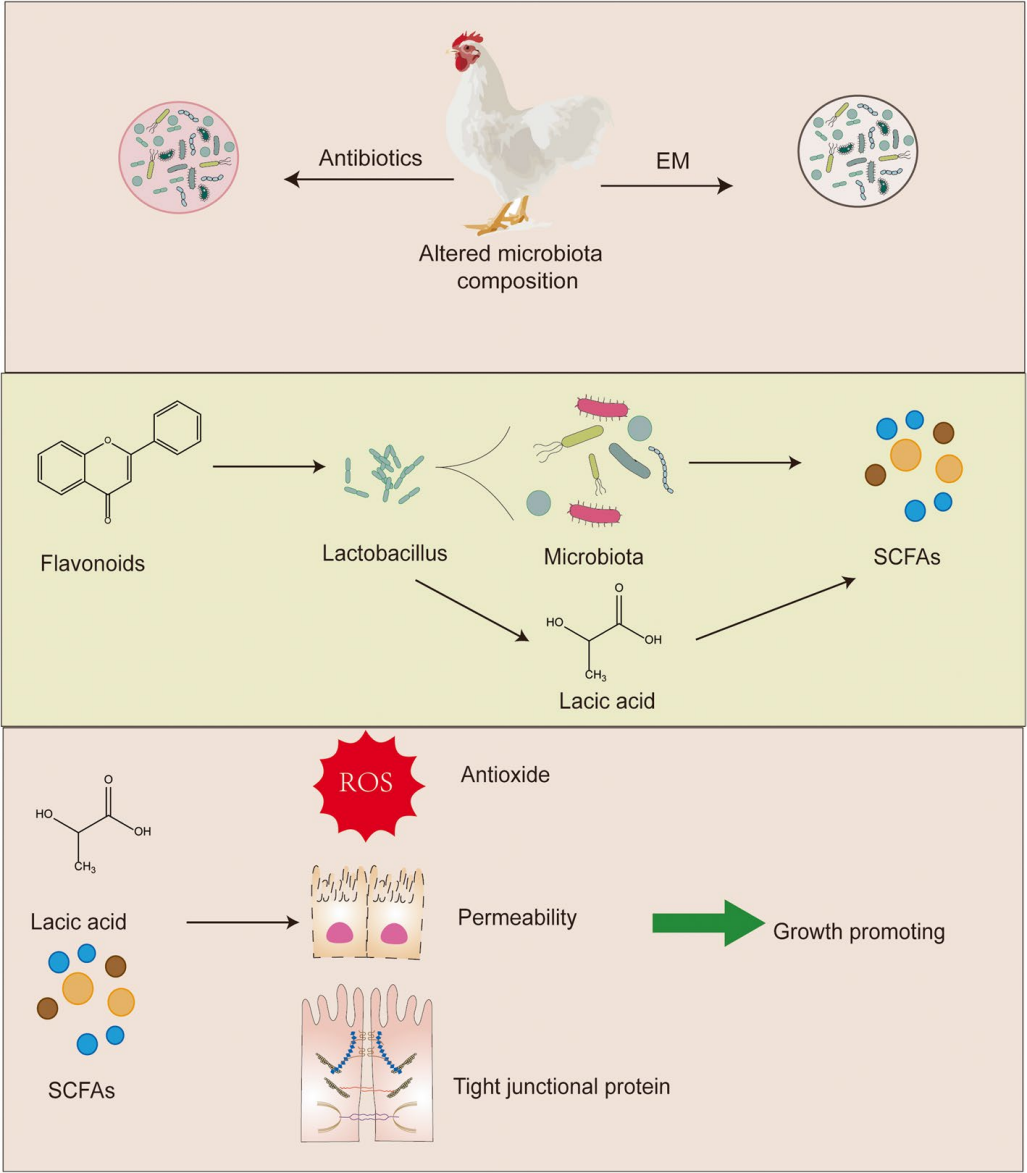

## Introduction

Many countries have banned the use of antibiotics in animal husbandry, and intestinal health is still a potential risk in livestock and poultry production [1, 2]. Due to the excessive growth rate of broilers, gut development is often imperfect. The health of broilers is severely challenged after antibiotics are discontinued, with impaired gut function, intestinal microbiota disturbances and oxidative stress contributing to poor broiler growth performance. Fortunately, plant natural products represent a promising source of antibacterial lead compounds that could help filling the absence of antibiotics in response to decline in production [3–5]. The use of natural bioactive compounds has attracted widespread attention for improving poultry health and performance [6].

Flavonoids are the major dietary polyphenols found in a variety of plant sources. Most flavonoids exist in nature as bound forms of flavonoid glycosides, in which one or more sugar moieties are bound to a phenolic or hydroxyl group at the C-3 position [7, 8]. Glycosidic bonds strongly affect the absorption of flavonoids, and most flavonoid glycosides can reach the gut as prototypes, where they may exert their physiological effects [9, 10]. Previous research has shown that flavonoids had a wide range of positive effects, such as improving gut morphology and function, and strengthening immunity [11]. Flavonoids are potential antibacterial compounds that can bind to phospholipids of bacterial membranes and lead to dissipation of proton motive force and metabolic perturbations, thereby exerting antibacterial effects [12]. Flavonoids can modulate the composition of the gut microbiome, inhibit the growth of various pathogens in the gut, and increase beneficial bacterial genera such as *Bifidobacterium* and *Lactobacillus* to shape the gut microbiome [13]. Meanwhile, flavonoids may reduce oxidative stress by downregulating nicotinamide adenine dinucleotide phosphate oxidase (NADPH) expression and improve intestinal tight junction barrier and structure, joint protection, and estrogenic effects [14]. However, the effects of flavonoids on broiler chicken gut microbes, gut barrier function and antioxidants require further study.

*Epimedium* (EM) is a common Chinese herbal medicine. Its main ingredients are prenyl flavonols, including icariin, epimedin A–C and baohuoside I, which are also functional ingredients considered by the Pharmacopoeia of the People’s Republic of China [8, 15]. They exhibit several biological activities, including antibacterial, antioxidant, antitumor and bone-protecting activities [15–18]. EM is also often allowed to be used as a feed additive in livestock and poultry breeding in many countries (such as China). However, the effect and how to exert the growth-promoting effect have not been systematically evaluated. In this study, we hypothesized that dietary supplementation of EM would positively alter microbial composition, beneficial metabolite content, gut barrier function and gut antioxidant capacity, thereby promoting improved growth performance in broilers. Therefore, this study aimed to investigate the effects of dietary EM supplementation on growth performance, gut barrier function and gut microbiota in broilers.

## Methods

### Chemicals and regents

For the chemical analysis, standards of epimedin A-C, icariin and baohuoside I were bought from Yuanye Bio-Technology Co., Ltd (Shanghai, China), which both had purity above 98%. Acetonitrile, methanol, and formic acid were purchased from Merck KGaA (Darmstadt, Germany).

### EM extracts

EM was purchased from Minxian (Gansu, China). The EM was extracted with water, separated with macroporous resin, eluted with ethanol, concentrated and spray dried. Ultra-high-performance liquid chromatography (UHPLC) analysis was conducted on the Agilent 1260 HPLC system (Agilent Technologies, Santa Clara, CA, United States) with an auto-sampler, online degasser, and a quaternary pump. A Waters C18 column (4.6 mm × 250 mm; 5 μm) was used for separating flavonoid substances. The mobile phase A was water, while phase B was acetonitrile, which the flow rate was 1 mL/min. The linear gradient elution program was: 0–15 min, 25% B; 15–23 min, 30% B; 23–33 min, 52% B; 33–40 min, 48% B, and the analyte injection volume was 5 µL, and the column temperature was 30 °C.

### Birds and experimental design

A total of 720 1-day-old broilers (AA+) were randomly divided into six groups (eight replicates, 15 birds per pen). The normal control (NC) group was fed the basal diet. The antibiotic (CTC) group was fed the basal diet supplemented with 75 mg/kg chlortetracycline. The remaining groups were fed the basal diet supplemented with 100, 200, 400 or 800 mg/kg EM (EM100, EM200, EM400 and EM800). Throughout the 42-d experiment, all birds were cultivated in single-layer hanging pens and provided free access to food and water, with 16 h of light per day. In the first week, the room temperature was 35 °C and was decreased by 2 °C each week. Experimental diets were formulated to meet nutrient requirement of broiler chicks (National Research Council, 1994 [19]; Ministry of Agriculture of China, 2004 [20]) (Table 1). Feed ingredient reference Tables of feed composition and nutritive values in China [21]. The measurement of total energy and crude protein in the nutrient composition refers to the recommendations of AOAC, and the remaining indicators are calculated values [22]. All experimental protocols were approved by the Animal Care and Use Committee of the Institute of Animal Science of the Chinese Academy of Agricultural Sciences (IAS2021-71), and the methods were carried out in accordance with the relevant guidelines and regulations.

**Table 1:** Composition and nutrient levels of experimental diet, % (air-dry basis)

Items	Starter phase (d 1 to 21)	Grower phase (d 22 to 42)
Ingredients		
Corn	55.03	58.58
Soybean meal	32.5	31.7
Fish meal	4.3	1.8
Soybean oil	4.2	4
Dicalcium phosphate	1.45	1.7
Limestone	1.34	1.15
*DL*-methionine	0.23	0.12
Sodium chloride	0.3	0.3
Choline chloride	0.15	0.15
Premix1	0.5	0.5
Nutrient levels		
Metabolizable energy, MJ/kg	12.77	12.98
Crude protein, %	21.59	20.02
Calcium, %	1.07	0.94
Total phosphorus, %	0.72	0.69
Available phosphorus, %	0.45	0.41
Lysine, %	1.21	1.08
Methionine + cysteine, %	0.94	0.78
Threonine, %	0.83	0.77
Tryptophan, %	0.25	0.24

Premix supplied per kg of diet: vitamin A, 10,000 IU; vitamin D_3_, 3200 IU; vitamin E, 15 IU; vitamin K_3_, 5 mg; thiamin, 2.1 mg; tiboflavin, 7 mg; vitamin B_12_, 0.02 mg; biotin, 0.17 mg; folic acid, 1.5 mg; iron (from FeSO_4_·H_2_O), 80 mg; copper (from Cu_2_(OH)_3_Cl), 20 mg; zinc (from ZnSO_4_·7H_2_O), 90 mg; manganese (from MnSO_4_ ·H_2_O), 80 mg; iodine (from KI), 0.4 mg; selenium (from Na_2_SeO_3_), 0.4 mg

### Sample collection

At 21 and 42 days of age, the body weight (BW), average daily gain (ADG), average daily feed intake (ADFI), and feed to gain ratio (F/G) of broilers from each cage were measured. Both feed intake and F/G were adjusted for mortality. Representative sample birds were killed at 21 and 42 d. Coagulation-promoting tubes were used to obtain serum; the blood was allowed to stand at room temperature for 4 h and centrifuged at 3000 × *g* for 15 min. The jejunal and ileum of selected birds were collected. Jejunal and ileal contents were collected for microbiota analysis. The mucosa was scraped by autoclaved blade after saline flush for gene expression analysis. All samples were stored at −80 °C.

### Serum antioxidant indexes

The serum antioxidant indexes were tested using commercial assay kits (Jiancheng Bioengineering Institute, Co. Ltd, Nanjing, China). The total antioxidant capacity (T-AOC) method based on ABTS (SKU: A015-2-1), glutathione peroxidase (GSH-Px) (SKU: A005-1-2), catalase (CAT) (SKU: A007-2-1), and malondialdehyde (MDA) method based on TBA (SKU: A003-1-2) were determined with Multiscan Spectrum (Tecan, Infinite 200 Pro, Switzerland).

### Intestinal permeability

The serum diamine oxidase (DAO) method based on UV colorimetry (SKU: A088-1-1) was tested using commercial assay kits (Jiancheng Bioengineering Institute, Co. Ltd, Nanjing, China). The serum endotoxins (SKU: ml059937) and *D*-lactate (SKU: ml367904) were tested using chicken ELISA kits (Shanghai Enzyme-linked Biotechnology Co. Ltd., China).

### Lactic acid and SCFAs analysis

The detection of lactic acid content was quantified by Agilent 6470 HPLC-MS (Agilent Technologies, Santa Clara, CA, United States). The stool content weighed about 0.5 g and was diluted with 2 mL ultrapure water, centrifuged (14,000 × *g*, 4 °C for 10 min), and filtered through a 0.22-µm microporous membrane. The liquid phase mass spectrometry system measured the concentration of lactic acid through the waters T3 column. Ultrapure water with 0.1% formic acid and methanol with 0.1% formic acid were selected as the mobile phase. In negative ion mode, the quantifier ion transition *m/z* 89.0 → 45.0 and the qualifier 89.0 → 43.1 were used with a fragmentor voltage of 50 V, collision energy of 8 V, and cell accelerator voltage of 5 V.

The detection of SCFAs content was quantified by means of gas chromatography Agilent 5975 C (Agilent Technologies, Santa Clara, CA, United States). Feces weighed approximately 0.5 g and were diluted with 2 mL ultrapure water and mixed well, then centrifuged (10,000 × *g*, 15 min at 4 °C). 900 µL supernatant being mixed with prepared 100 µL ice-cold 25% (w/v) metaphosphoric acid solution at 4 °C for 4 h. Centrifuge the mixture (10,000 × *g*, 15 min) and filter the supernatant using a 0.45-µm microporous membrane. The gas chromatography system measured concentrations of acetate, propionate, isobutyrate, butyrate, isovalerate and valerate by DB-FFAP column (30 m × 250 μm × 0.25 μm). The carrier gas was N_2_ (12.5 Mpa, 0.8 mL/min). The temperature of the FID (flame ionization detector) detector was 280 °C and that of column was heated from 60 to 220 °C at a rate of 20 °C/min.

### The 16S rRNA high-throughput sequencing

Total genomic DNA from the jejunal and ileal samples was extracted by QIAamp DNA Stool Mini Kit (Qiagen, Hilden, Germany). DNA concentration and integrality were detected by NanoDrop Spectrophotometer (Thermo Scientific, Wilmington, DE, USA) and agarose gel electrophoresis, respectively. DNA concentration of each cecal content was diluted to 10 ng/µL using double-distilled water. The V3–V4 region of 16S rDNA was amplified using the following specific primers (338F: 5´-ACTCCTACGGGAGGCAGCAG-3´; 806R: 5´-GGACTACHVGGGTWTCTAAT-3´). Purified amplicons were pooled in equal amounts and paired-end sequenced (2 × 250 bp) on an Illumina MiSeq platform at Majorbio Bio-Pharm Technology Co. Ltd. (Shanghai, China).

The raw reads were deposited into the NCBI Sequence Read Archive database (Accession Number: PRJNA839558). Fastp software (version 0.19.6) was used for quality control of sequencing results, and raw sequencing data were presented in fastq format. Flash software (version 1.2.11) was used to assemble the paired-end reads. Noise reduction of the sequences and removal of reads with chimera were performed with Qiime2 software. The clustering of operational taxonomic units (OTUs) was generated from clean reads using Uparse software (version 11) with a 97% similarity requirement. The entire representative reads selected by the Qiime2 package were annotated with the SILVA database (Version 138) using a RDP classifier (with a 70% confidence threshold). According to the OTU clustering, the alpha-indexes were used to describe the diversity of jejunal and ileal microbial species within each group. Partial least squares discriminant analysis (PLS-DA) was conducted to compare the bacterial community structures across all samples. Moreover, the significance of differentiation of microbial structure among groups was statistically tested by analysis of similarity. The linear discriminant analysis (LDA) was coupled with effect size measurements (LEf-Se) to distinguish the bacteria between all the treatments, the LDA score was set at two.

### Gene expression

Total RNA from ileal mucous membrane was extracted by TRIzol reagent (Takara, Dalian, China). Primer premier 6 was used to customize primers the shown in Table 2. One microgram of total RNA was used to synthesize cDNA using the PrimeScript RT Reagent Kit (Takara). ABI 7500 Real-time PCR Instrument was used for real-time PCR (Thermo Fisher Scientific, Waltham, MA, USA). The PCR system comprised 1 µL of 5× diluted cDNA, 0.4 µL each of 10 µmol/L forward and reverse primers, 5 µL TB Green Premix Ex Taq II (Takara), 0.2 µL ROX Reference Dye II (Takara) and 3 µL DNase-free distilled water. The PCR amplification was in two stages including 95 °C for 30 s, followed by 40 cycles of 95 s owed by es incl for 34 s. The specificity of the primers was examined by melting curve analysis. The gene β-actin was selected as a reference, which was used to normalize the relative expression of genes of interest by the 2^−ΔΔCT^ method.

**Table 2:** The primers for quantitative real-time PCR

Gene	Primer sequences (5' to 3')	Accession number	Length, bp
*SOD1*	F:CAAAGGGAGGAGTGGCAGAAGTAGA	NM_205064.1	160
	R:CGAGGTCCAGCATTTCCAGTTAGTTT		
*CAT*	F:CGTATTCAGGCACTGCTGGACAA	NM_001031215.2	83
	R:CGAGAAGTGGCTTGCGTGTATGT		
*GPX1*	F:GCAAAGTGCTGCTGGTGGTCAA	NM_001277853.2	166
	R:ATCTCCTCGTTGGTGGCGTTCT		
*Occlaudin*	F:GCAGATGTCCAGCGGTTACTACTAC	NM_205128.1	177
	R:GCGAAGAAGCAGATGAGGCAGAG		
*Claudin-1*	F:GGAGGATGACCAGGTGAAGAAGATG	NM_001013611.2	115
	R:CCGAGCCACTCTGTTGCCATAC		
*ZO-1*	F:GGTGCTTCCAGTGCCAACAGAA	XM_040680632.1	182
	R:GCCAACCGTAGACCATACTCTTCATT		
*β-actin*	F:CTGGTATTGTGATGGACTCTGGTGATG	NM_205518.1	162
	R: TGGTGGTGAAGCTGTAGCCTCTC		

### Co-occurrence network analysis

We calculated the Spearman’s rank correlation coefficient and *P*-value through the R package of Hmisc according to the relative abundance distribution of the genera. The combination of correlation coefficient *ρ* > 0.3 or *ρ* <−0.3 and *P* < 0.01 was screened-out. Networks were constructed using the method implemented in Cytoscape (version 3.8.1).

### Statistical analysis

The data in a completely randomized design were analyzed using the one-way ANOVA procedure of SAS 9.4 software (SAS Institute, Cary, NC, USA), with cage as the experimental unit for analyzing growth performance and each selected bird as the experimental unit for other parameters. *P* < 0.05 was considered statistically significant. All data were expressed as means and pooled SEM. The heat map analysis utilized R package pheatmap, and GraphPad Prism 8.0 (GraphPad Software, San Diego, CA, USA) was used to produce the histogram.

## Results

### Major composition of EM

The major flavonoid constituents of EM were analyzed using HPLC. The major constituents were icariin (23.37 ± 0.87 g/100 g), epimedin A (2.82 ± 0.09 g/100 g), epimedin B (7.45 ± 0.26 g/100 g), epimedin C (11.65 ± 040 g/100 g), and baohuoside I (0.77 ± 0.01 g/100 g).

### Growth performance

The effects of EM on growth performance of broilers are shown in Table 3. Compared with the NC group, ADFI in the CTC group increased significantly during 1–21 d (*P* < 0.01), and there was no significant difference between the CTC and EM treatment groups. Compared with the NC group, ADG in the CTC and EM200 groups increased significantly at 21 and 42 d (*P* < 0.01 and *P* < 0.05, respectively). The feed conversion ratio (FCR) in the EM100 group was significantly lower than that in the NC group during 1–21 d (*P* < 0.05).

**Table 3:** Effect of dietary EM on growth performance of broilers

Items	Treatments						SEM	*P*-value
	NC	CTC	EM100	EM200	EM400	EM800		
Starter phase (d 1 to 21)								
Initial weight, g	39.65	39.46	39.61	39.29	39.63	39.01	0.47	0.727
BW, g	646.88	677.50	654.38	663.98	655.94	650.78	11.29	0.113
ADFI, g	37.85^a^	39.98^b^	38.57^ab^	39.23^a^^b^	38.69^ab^	38.29^ab^	0.48	0.002
ADG, g	28.62^a^	30.52^b^	29.51^ab^	29.99^b^	29.45^ab^	29.21^ab^	0.45	0.004
FCR	1.33^b^	1.30^ab^	1.30^a^	1.31^ab^	1.31^ab^	1.31^ab^	0.01	0.049
Starter phase (d 22 to 42)								
BW, g	1986.11	2139.13	2069.81	2107.34	2066.41	2091.36	52.31	0.114
ADFI, g	101.21	108.78	102.41	105.26	102.72	100.05	3.02	0.078
ADG, g	63.61	71.04	66.44	68.02	66.8	67.56	2.28	0.071
FCR	1.56	1.54	1.56	1.56	1.55	1.57	0.02	0.834
Starter phase (d 1 to 42)								
ADFI, g	69.09	73.31	71.26	72.88	70.52	68.99	3.02	0.078
ADG, g	45.65^a^	49.06^b^	48.28^a^^b^	49.13^b^	47.59^a^^b^	46.17^a^	2.28	0.031
FCR	1.51	1.49	1.48	1.48	1.48	1.5	0.02	0.830

^a,b^Means that different superscripts in the same row differ significantly (*n* = 8, *P* < 0.05)

* BW* body weight, *ADG *average daily gain, *ADFI *average daily feed intake, *FCR *feed conversion ratio (feed:gain, g:g)

**Table 4:** Effect of dietary EM on intestinal permeability of broilers

Items	Treatments						SEM	*P*-value
	NC	CTC	EM100	EM200	EM400	EM800		
21 d								
DAO, U/L	10.63^bc^	11.18^c^	9.58^abc^	9.12^ab^	8.93^a^	10.11^abc^	0.49	0.001
LPS, EU/L	0.4^bc^	0.42^c^	0.38^ab^	0.37^a^	0.37^a^	0.4^bc^	0.01	<0.001
*D*-lactic, µmol/mL	88.74^c^	92.41^c^	83.24^bc^	64.5^a^	74.24^ab^	85.37^bc^	3.90	<0.001
42 d								
DAO, U/L	7.38^ab^	8.02^b^	6.72^a^	6.9^ab^	6.76^a^	6.86^ab^	0.42	0.023
LPS, EU/L	0.45^c^	0.44^c^	0.39^b^	0.37^ab^	0.35^a^	0.36^ab^	0.01	<0.001
*D*-lactic, µmol/mL	93.16^c^	94.45^c^	75.31^a^	77.33^ab^	80.9^ab^	86.04^bc^	3.39	<0.001

^a,b,c^ Means with different superscripts in the same row differ significantly (*n* = 8, *P* < 0.05)

*DAO* diamine oxidase, *LPS *lipopolysaccharide

### Serum antioxidant indexes

**Fig. 1:**
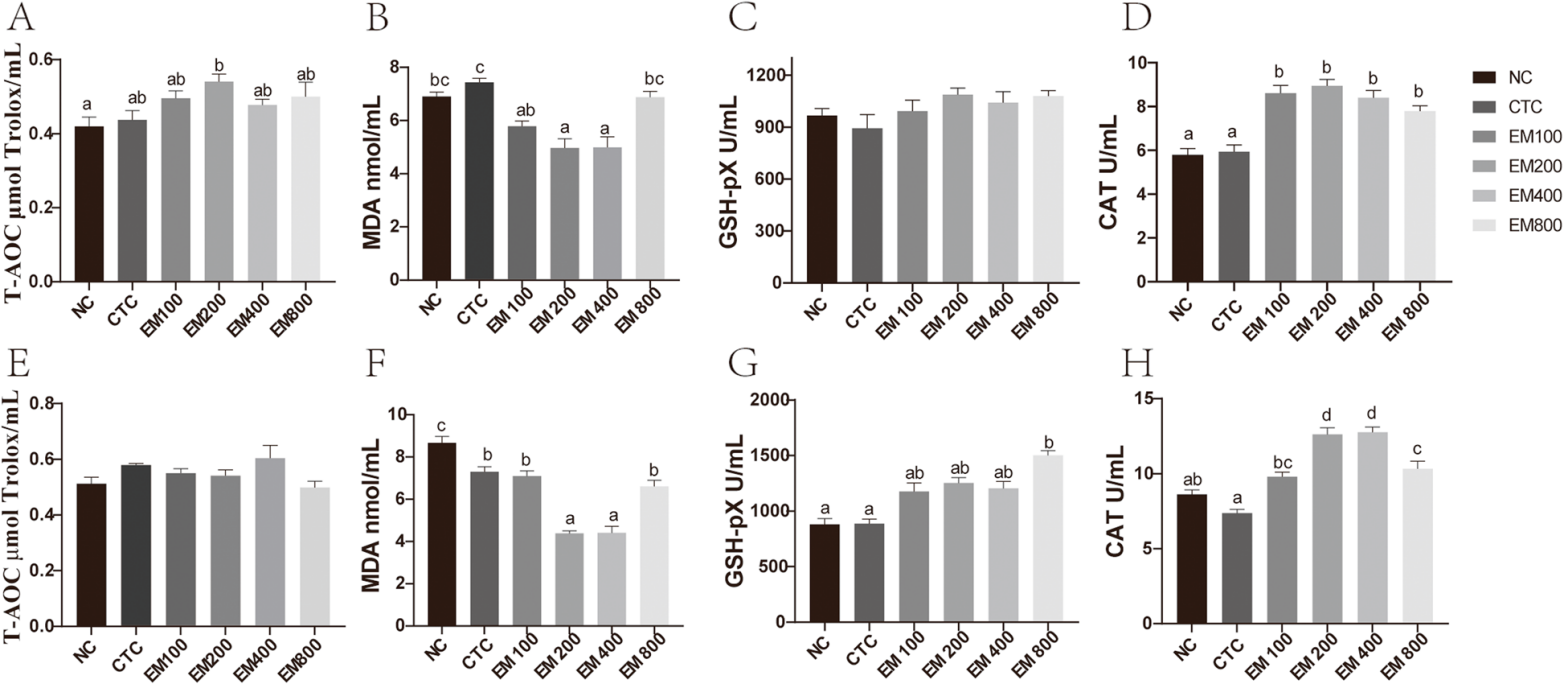
Effects of EM on serum T-AOC (**A**, **E**) and concentration of MDA (**B**, **F**), GSH-Px (**C**, **G**), and CAT (**D**, **H**) in broilers on 21 d (**A**–**D**) and 42 d (**E**–**H**)

The effects of EM on the serum antioxidant parameters are shown in Fig. 1. Compared with the NC group, T-AOC at 21 d was significantly increased in the EM200 group (*P* < 0.05). Compared with the CTC group, the dietary supplementation of 100, 200 and 400 mg/kg EM significantly reduced the 21-d serum MDA content (*P* < 0.001). Dietary supplementation with antibiotic and EM significantly reduced the 42-d serum MDA content (*P* < 0.001), and the MDA content of the EM200 and EM400 groups was significantly lower than that of the CTC, EM100 and EM800 groups (*P* < 0.001). Compared with the NC and CTC groups, the EM800 group had a significantly higher content of GSH-Px at 42 d (*P* < 0.001). Supplementation with EM significantly increased serum CAT at 21 d (*P* < 0.001), and the serum CAT of the EM200, EM400 and EM800 groups was significantly higher than that of the CTC group at 42 d (*P* < 0.001).

### Intestinal permeability

The effects of EM on intestinal permeability of broilers are shown in Table 4. Compared with the NC and CTC groups, serum DAO concentration at 21 d in the EM400 group was significantly lower (*P* < 0.01), and serum DAO concentration in the EM200 group was significantly lower than that in the CTC group at 42 d (*P* < 0.05). Compared with the NC and CTC groups, serum LPS concentration in the EM200 and EM400 groups at 21 d was significantly reduced (*P* < 0.001). Addition of EM to the diet significantly reduced serum LPS concentration at 42 d (*P* < 0.001). Compared with the NC and CTC groups, the EM200 and EM400 groups had significantly reduced serum *D*-lactic acid concentration at 21 and 42 d (*P* < 0.001 and *P* < 0.001, respectively).

**Fig. 2:**
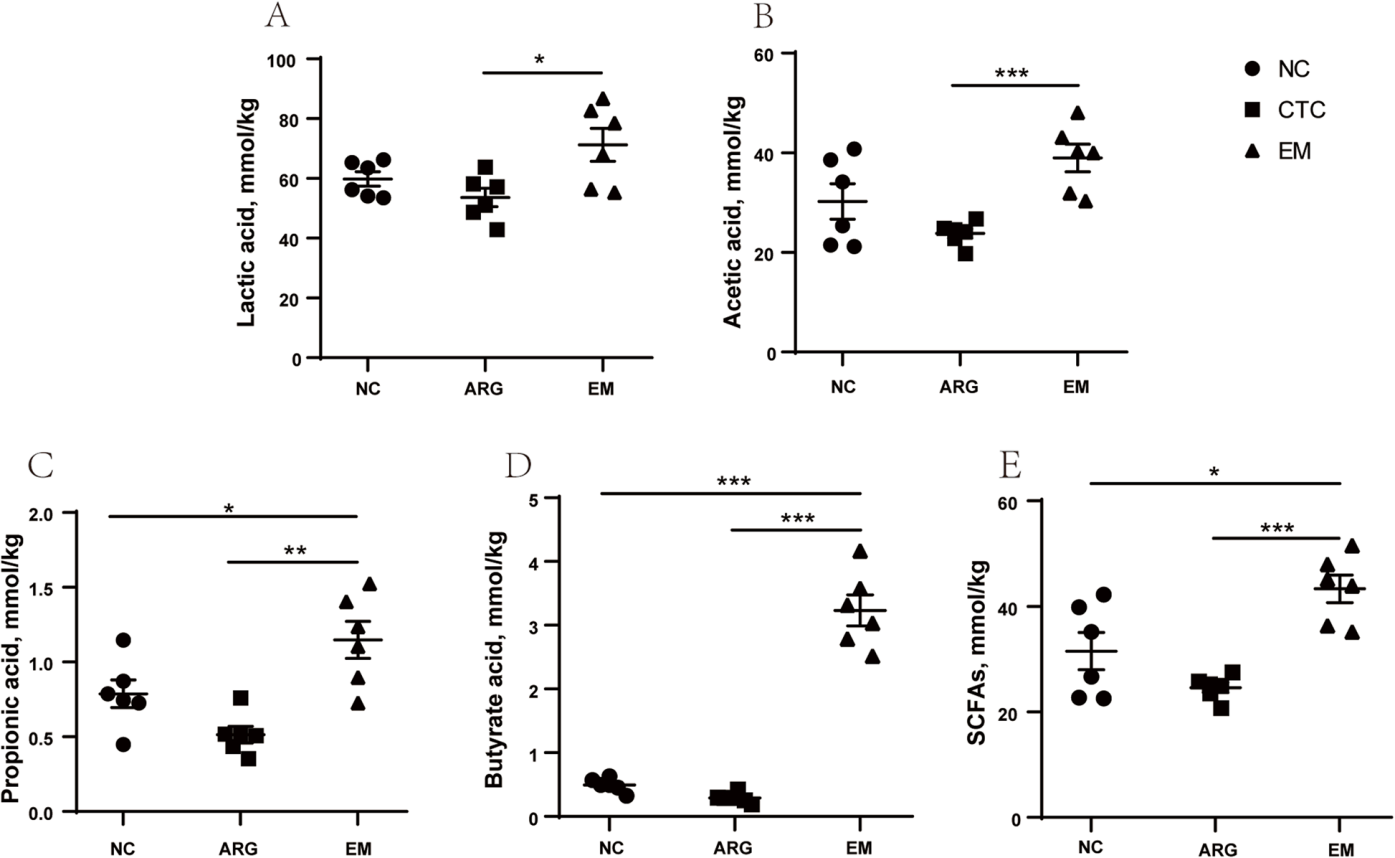
Effects of EM on fecal concentrations of lactic acid (**A**), acetic acid (**B**), propionic acid (**C**), butyrate acid (**D**) and total SCFAs (**E**) on 42 d. Significant difference was recorded by 0.01< *P* ≤ 0.05*, 0.001< *P* ≤ 0.01^**^, *P* ≤ 0.001^***^. SCFAs, short-chain fatty acid

### Lactic acid and SCFAs analysis

The concentration of lactic acid and SCFAs in feces is shown in Fig. 2. The concentration of lactic acid and acetic acid in the feces of the EM200 group was significantly higher than that in the CTC group (*P* < 0.05 and *P* < 0.001, respectively). Compared with the NC and CTC groups, the fecal propionic acid, butyric acid and SCFAs in the EM group were significantly increased (*P* < 0.01, *P* < 0.001 and *P* < 0.001, respectively).

**Fig. 3:**
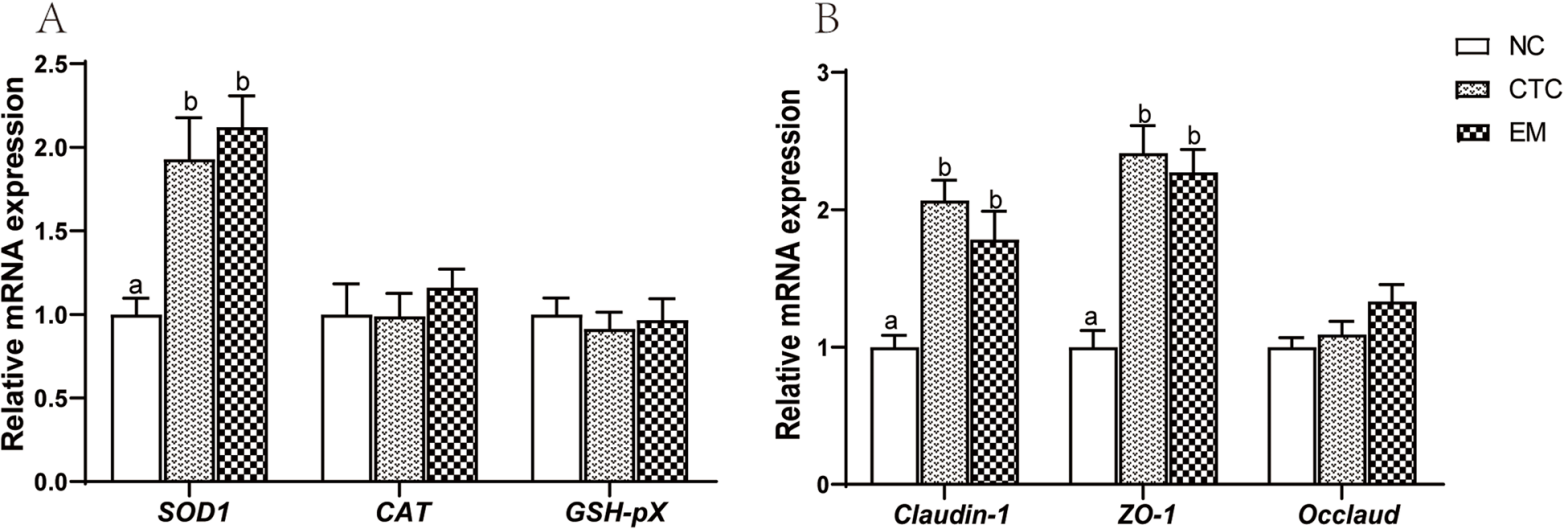
Effects of EM on the relative mRNA expression of ileal antioxidant enzymes and barrier function genes of broilers on 42 d. ^a,b^Means with different superscripts in the same index differ significantly (*n* = 8, *P* < 0.05)

### Gene expression

The effects of 200 mg/kg EM on ileal antioxidant capacity and barrier function were investigated. mRNA expression of antioxidant genes (*SOD1*, *CAT* and *GSH-Px*) and intestinal barrier genes (*Occludin*, *Claudin-1* and *ZO-1*,) is shown in Fig. 3. Compared with the NC group, expression of *Claudin-1* and *ZO-1* genes was significantly upregulated in the CTC and EM200 groups (*P* < 0.001, *P* < 0.001, *P* < 0.001, *P* < 0.01). Dietary supplementation of chlortetracycline and 200 mg/kg EM significantly increased ileal expression of *SOD1* gene (*P* < 0.01).

**Fig. 4:**
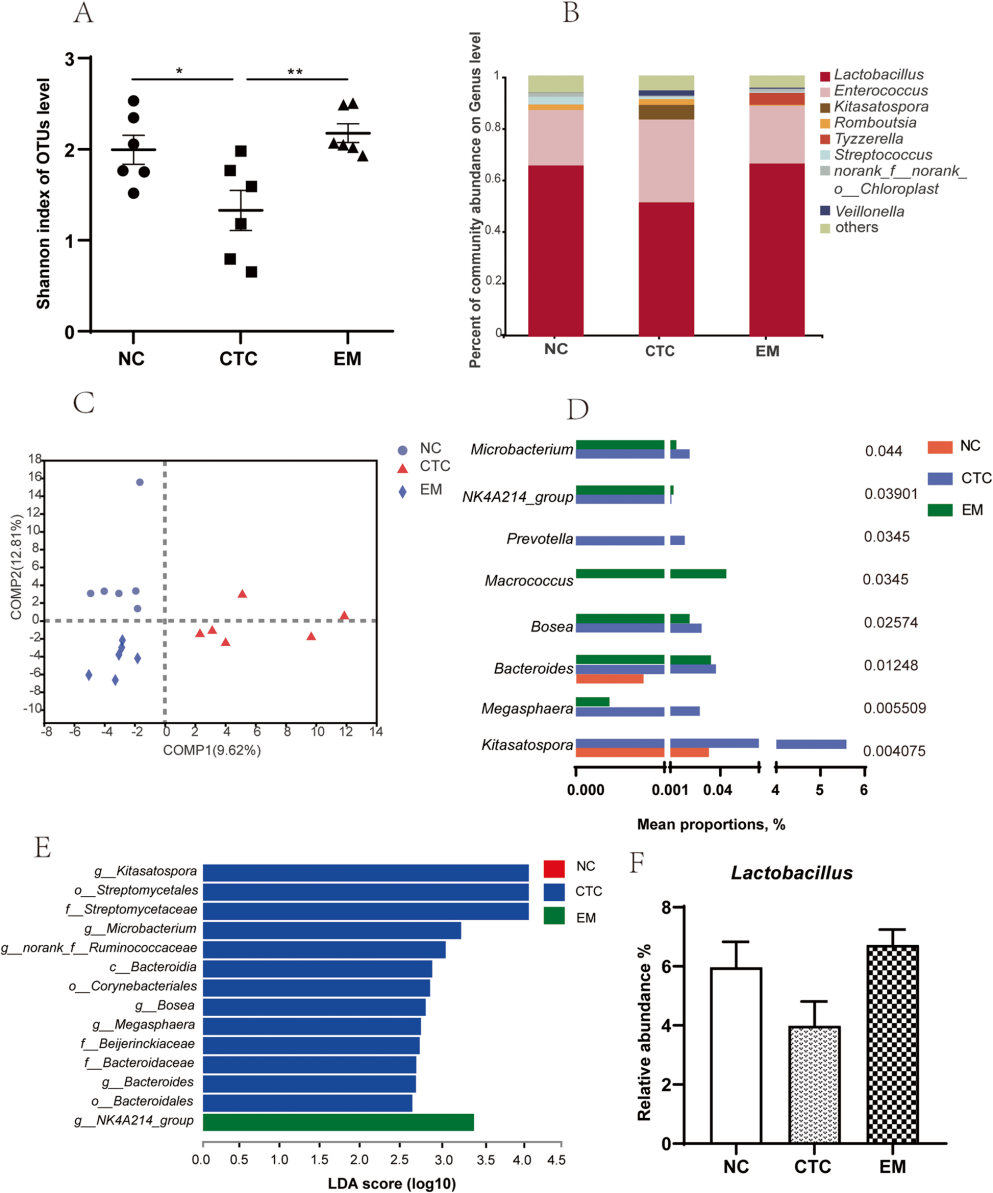
Effects of EM on jejunal microbiota diversity (**A**) and microbiota composition at genus level (**B**) of broilers on 42 d. (**C**) Comparison of samples between multiple groups based on PLS-DA. (**D**) Differential bacteria at genus level by Kruskal–Wallis H test. Effects of EM on linear discriminant analysis effect size (LEfSe) to detect the most significantly abundant jejunal microbiota of broilers on 42 d. (**E**) LDA score generated for differentially abundant microbiota (LDA > 2, *P* < 0.05). Significant defference was recorded by 0.01 < *P* ≤ 0.05^*^, 0.001 < *P* ≤ 0.01^**^, *P* ≤ 0.001^***^

### Jejunal microbial analysis

Compared with the NC and EM200 groups, the α-diversity estimators (Fig. 4A) of microbial richness, including Shannon index, in the jejunal were significantly reduced in the CTC group (*P* < 0.05). To analyze microbial composition, genus was selected as the taxonomic level. *Lactobacillus*, *Enterococcus*, *Kitasatospora*, *Romboutsia*, *Tyzzerella*, *Streptococcus*, *norank_f_norank_o_Chloroplast* and *Veillonella* were the main genera (> 1%) in the jejunal of broilers, and *Lactobacillus* and *Enterococcus* were the dominant genera, while the relative abundance of *Lactobacillus* was higher than that of *Enterococcus* in all groups (Fig. 4B). Microbial profile was clustered using PLS-DA (Fig. 4C), by which jejunal microbial communities of broilers were distributed in three detached clusters. Dietary supplementation with chlortetracycline and EM exerted a different effect on microbial composition. Kruskal-Wallis H test was used to test the significance of differences between groups at the genus level (Fig. 4D). Compared with the NC group, the relative abundance of *Microbacterium*, *NK4A214_group*, *Bosea*, *Bacteroides* and *Megasphaera* in the CTC and EM groups was significantly increased (*P* < 0.05, *P* < 0.05, *P* < 0.05, *P* < 0.05 and *P* < 0.01, respectively). The relative abundance of *Macrococcus* was significantly increased in the EM groups, but the relative abundance of *Kitasatospora* was decreased (*P* < 0.05 and *P* < 0.01, respectively). The relative abundance of *Prevotella* in the CTC group increased significantly (*P* < 0.05). Furthermore, linear discriminant analysis effect size (LEfSe) analysis (Fig. 4E) was explored to identify significant taxa in phylotypes. Seven genera, three families and three orders were detected with LDA threshold > 2. The genera *Kitasatospora*, *Microbacterium*, *norank_f_Ruminococcaceae*, *Bosea*, *Megasphaera* and *Bacteroides*, the order *Streptomycetales*, *Corynebacteriales* and *Bacteroidales*; and families *Streptomycetaceae*, *Beijerinckiaceae* and *Bacteroidaceae* were biomarkers in the CTC group. The jejunals microbial community of broiler was characterized by *NK4A215_group* as a biomarker in the EM groups (*P* < 0.05). *Lactobacillus* was the predominant genus in the jejunal. The relative abundance of *Lactobacillus* in the EM groups was higher than that in the CTC group (*P* > 0.05).

### Ileal microbial analysis

**Fig. 5:**
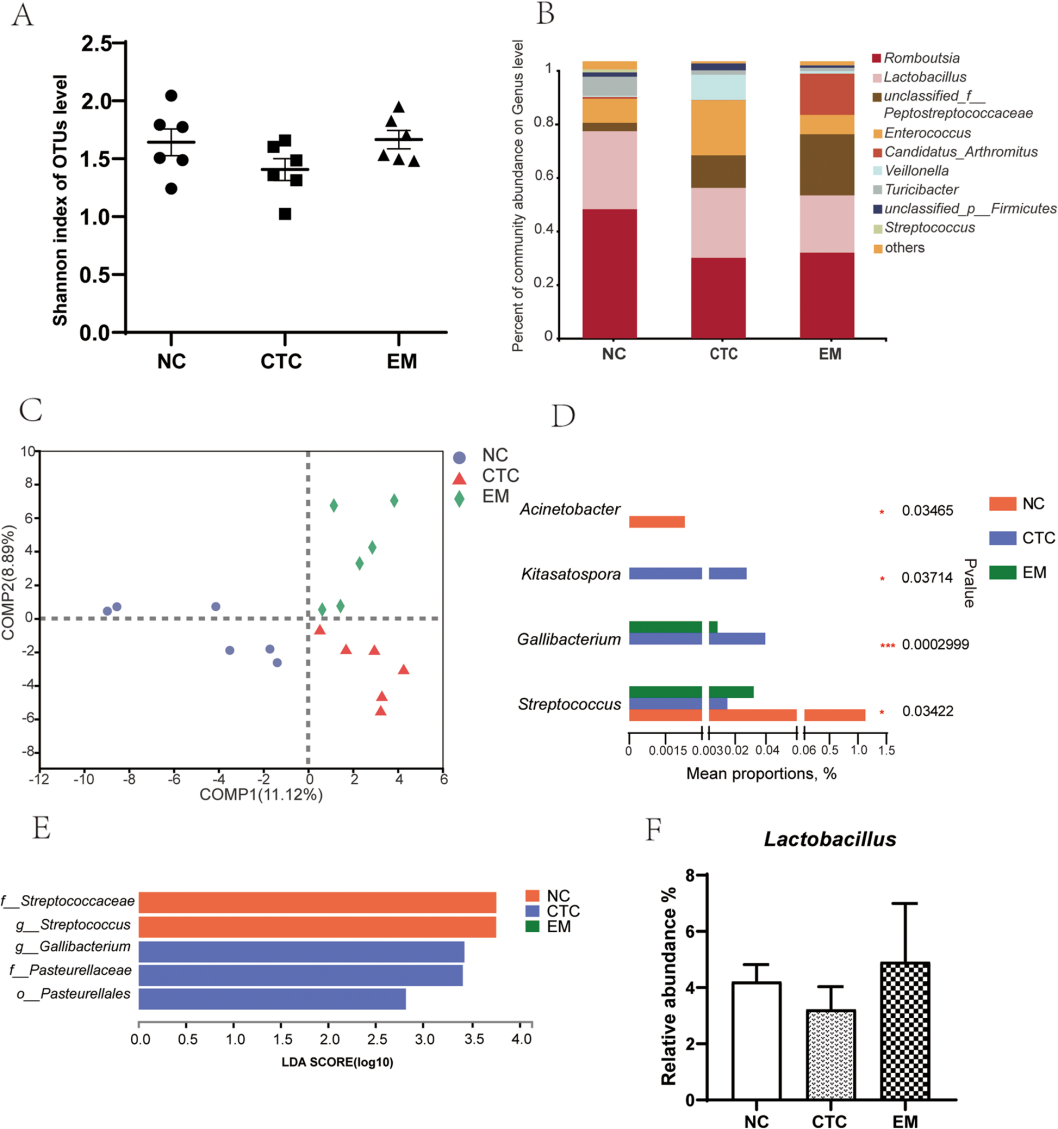
Effects of EM on ileal microbiota diversity (**A**) and microbiota composition at genus level (**B**) of broilers on 42 d. **C** Comparison of samples between multiple groups based on PLS-DA. **D** Differential bacteria at genus level by Kruskal–Wallis H test. Effects of EM on linear discriminant analysis effect size (LEfSe) to detect the most significantly abundant ileal microbiota of broilers on 42 d. **E** LDA score generated for differentially abundant microbiota (LDA > 2, *P* < 0.05). Significant difference was recorded by 0.01 < *P* ≤ 0.05^*^, 0.001 < *P* ≤ 0.01^**^, *P* ≤ 0.001^*^^**^^*^

Dietary supplementation with chlortetracycline and 200 mg/kg EM had no significant effect on the α-diversity of ileal microbes (Fig. 5A), but the α-diversity of the CTC group, including the Shannon index, was the lowest among the three groups (*P* > 0.05). Genus was selected as the taxonomic level for analysis of microbial composition. The ileal microbial communities of all groups were predominated by *Romboutsia*, *Lactobacillus* and *unclassified_f_Peptostreptococcaceae* (Fig. 5B), and their content exceeded 60%. PLS-DA was used to cluster the microbiota (Fig. 5C), and the microbiota were divided into three independent groups. There were significant differences in microbial communities, indicating that dietary supplementation with chlortetracyline and 200 mg/kg EM had different effects on microbial composition. At the genus level, the Kruskal-Wallis H test was used to test the significance of differences between groups (Fig. 5D). Compared with the NC group, the relative abundance of *Acinetobacter* and *Streptococcus* in the CTC and EM groups was significantly reduced (both *P* < 0.05), the relative abundance of *Gallibacterium* was significantly increased (*P* < 0.05). The CTC group had a significantly increased relative abundance of *Kitasatospora*. LEf-Se analysis (Fig. 5E) was used to identify important taxa. LDA threshold > 2 of two genera, two families and one order was detected. The family Streptococcaceae and the genus *Streptococcus* were biomarkers in the NC group. The genus *Gallibactetium*, family Pasteurellaceae and order Pasteurellales were the ileal microbial community biomarkers in the CTC group. The relative abundance of *Lactobacillus* in the EM group was higher than that in the CTC group (*P* > 0.05).

### Co-occurrence network analysis

**Fig. 6:**
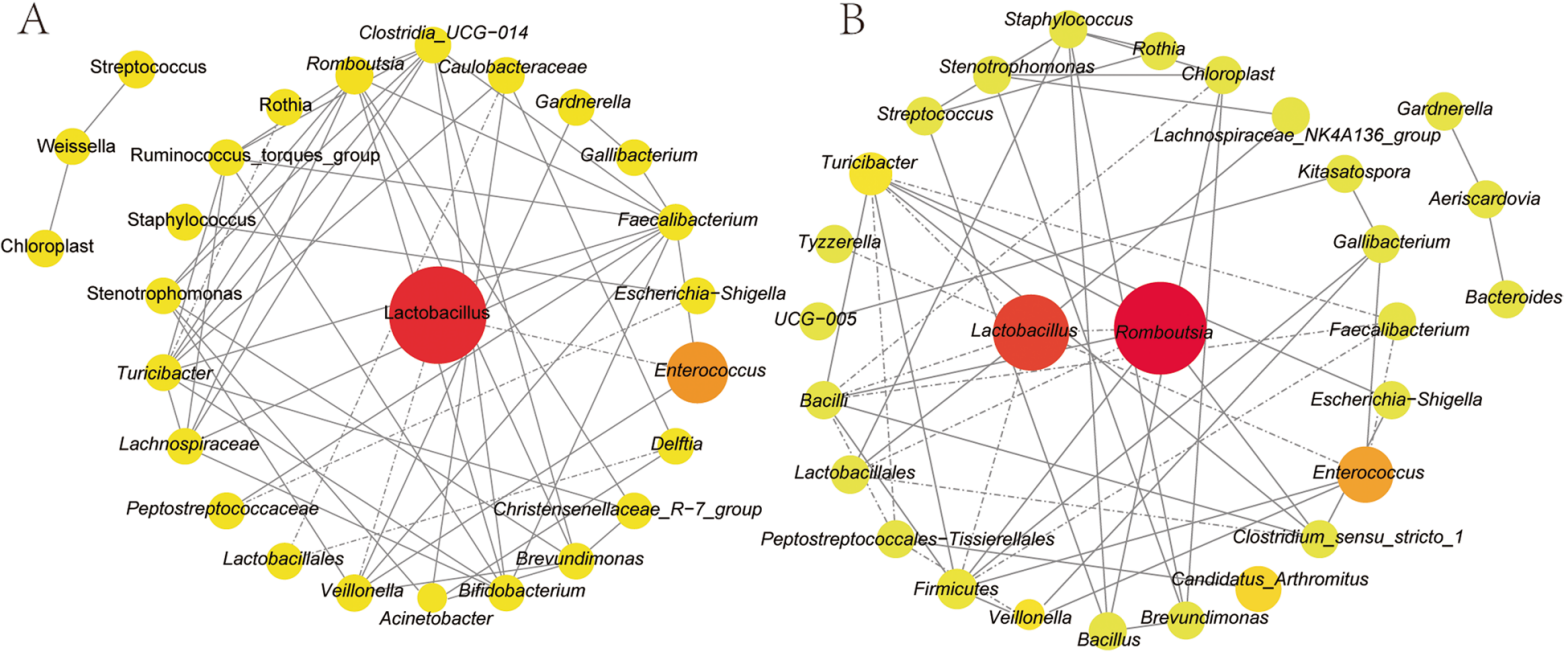
Effects of EM on co-occurrence network analysis of jejunal (**A**) and ileal (**B**) microbiota communities correlation of top 30 relative abundance genera

The interaction between the top 30 genera with relative abundance in the jejunal (Fig. 6 A) and ileum (Fig. 6B) was analyzed by co-occurrence network. *Lactobacillus* was the main genus and core genus in the jejunal and ileum. *Lactobacillus* mainly regulates the microorganisms of the jejunal by negatively regulating *Enterococcus* and *Veillonella*. Moreover, *Lactobacillus* mainly negatively regulated *Romboutsia*, *Turicbacter*, *unclassified_c_Bacilli* and *unclassified_p_Fimicutes* genera, and positively regulated *Lachnospiraceae_NK4A136_group* and *unclassified_o_Lactobacillales* in the ileum.

## Discussion

Beneficial effects of natural flavonoids on performance have already been described in poultry [23, 24]. In our study, dietary EM supplementation improved broiler growth performance by increasing ADG, and enhanced ileal antioxidant capacity and gut barrier function, suggesting that growth promotion was closely related to gut health. This study showed that 200 mg/kg EM increased serum GSH-Px and CAT enzyme activity and decreased serum MDA content to improve broiler health, along with increased expression of ileal *SOD1* antioxidant enzyme genes and *Claudin-1* and *ZO-1* barrier genes. Our results are partially supported by Yang et al. [25], who observed that EM flavonoids considerably affected antioxidant defense, including improving CAT and GSH-Px activities. Caco-2 cells supplemented with EM flavonoids increased transepithelial electrical resistance across cell monolayers, suggesting that EM flavonoids can increase tight junction integrity, and these changes in intestinal barrier function were caused by modification of the *ZO-1*, *ZO-2*, *Occludin* and *Claudin-1* genes [26]. The intestinal mucosal surface is in prolonged contact with luminal bacteria, bacterial products, and dietary antigens, which reinforces the need for a highly selective barrier. Tight junction proteins constitute the physical barrier of the gut and regulate its permeability, playing a crucial role in gastrointestinal protection [27]. There is growing evidence that flavonoids and their metabolites have health-promoting properties on gut barrier function, including protection of barrier permeability, positive regulation of gut microbiota, and inhibition of oxidative stress and inflammation in the gut lumen [28]. This is consistent with our findings that dietary EM supplementation can suppress oxidative stress in the intestinal lumen by increasing the expression of tight junction proteins and improving permeability.

Because the beneficial effects of EM supplementation were mainly seen in the EM200 group, the modulating effect of EM on gut microbes was assessed in the control, CTC and EM200 groups. Gut microbiota diversity based on OTU number was altered by treatment. The addition of chlortetracycline to the jejunal decreased Shannon index, whereas addition of EM increased Shannon index, suggesting that EM positively regulated species richness and chlortetracycline decreased species richness. In a previous study, Loo et al. reported that flavonoids have beneficial effects on the diversity of microbial colonies [29]. The diversity of gut microbes is determined by the composition and relative abundance of different species, and is influenced by multiple factors, such as age, health status, host genetics, dietary patterns, and environment [30]. Commercial incubation processes attenuate the influence of genetic factors on the gut microbiome of broilers, making environmental and dietary factors more important. The community structure of all groups was compared by PLS-DA analysis. The PLS-DA plots showed that the jejunal and ileal microbial communities were distributed in three separate clusters, suggesting that the effects of different treatments on microbial composition were different.

EM can improve the microbiome and thus promote gut health. The gut microbiota is an important barrier in the immune system; it affects the host’s immune and metabolic systems and is significantly associated with host health [31]. In the present study, *Lactobacillus* was the dominant genus, which was in line with the study of Feng et al. [32]. The improvement in the relative abundance of *Lactobacillus* could bring benefits to normal colonization of microbial communities. In the present study, the CTC group was enriched for the antibiotic-producing genus *Kitasatospora* and order Streptomycetales in the jejunal. *Kitasatospora* produces a variety of antibiotics [33]. It is speculated that the production of natural antibiotics amplifies the antibacterial and anti-inflammatory effects of administered antibiotics, which may be responsible for the growth-promoting effects of antibiotics. Compared with the CTC group, the addition of EM decreased the relative abundance of some harmful genera, such as *Microbacterium* and *Bacteroides* in the jejunal and *Gallibacterium* in the ileum, which are species that seriously threaten animal health [34]. *Bacteroides* was found among all anaerobic pathogens. It has the most antibiotic resistance mechanisms and the highest resistance rates, and is a clinically important pathogen, resulting in high mortality [35]. *Gallibacterium* is a common pathogen in poultry and severely affects the growth performance and mortality of poultry [36]. EM supplementation in the jejunal increased the abundance of some beneficial bacteria, such as *g_NK4A214_group* and *Lactobacillus*, which help maintain the overall microbial structure. *Lactobacillus* is the main genus that produces lactic acid. A recent study showed that *g_NK4A214_group* was positively correlated with isobutyrate and isovalerate concentrations [37]. Therefore, dietary 200 mg/kg EM regulated balance of gut microbiota may be a biomarker for estimating luminal function and health in broilers.

Changes in the relative abundance of gut microbes were clearly correlated with their metabolites. It has been shown that flavonoids could exert a prebiotic mechanism to enhance the relative abundance of *Lactobacillus* and *Bifidobacterium* [38]. Consistent with our study, the relative abundance of *Lactobacillus* in the jejunal and ileum was increased in the EM200 group compared with the CTC group. *Lactobacillus* is an important bacterium in the gut with a wide range of benefits, including inhibiting pathogen customization and positively improving host gut health [39, 40]. As one of the main genera in the chicken intestine, the main product of lactic acid bacteria is lactic acid. Lactic acid is an important compound linking food and gut microbes [41]. Lactic acid can also be used by sunlight bacteria to produce butyric acid through a cross-feeding mechanism. Our study also showed that dietary 200 mg/kg EM significantly increased fecal lactate and SCFAs content. Therefore, 200 mg/kg EM had a bidirectional regulatory effect with the microbiota and selectively promotes the growth and reproduction of beneficial gut bacteria, and metabolites of beneficial bacteria can enhance gut health.

In the present study, *Lactobacillus* was at the center of the co-occurrence network in both jejunal and ileal microbes. EM (200 mg/kg) could induce *Lactobacillus* to produce more lactic acid to regulate other bacterial genera in the gut, and further metabolism of lactic acid into SCFAs can have beneficial effects on gut health. Butyric acid is not only an important energy source for gut microbes, but it can also improve the function of the gut barrier [42]. In the gut, *Lactobacillus* can competitively inhibit some pathogenic bacteria, such as *Enteroccoccus*, *Veillonella* and *Romboutsia*, which are negatively correlated with these genera. *Lactobacillus* and SCFAs producers such as *unclassified_o_Lactobacillales* and *Lachnospiraceae_NK4A136_group* with anti-inflammatory effects were positively correlated with each other. The results are consistent with the study by Huang et al., who revealed that *Lactobacillus* predominated in the foregut of broilers, and that it was positively correlated with other SCFAs producers such as *Clostridium*, *Butyricicoccus* and *Faecalibacterium* [43]. Therefore, it can be speculated that dietary 200 mg/kg EM may promote gut health by altering gut microbes to produce more lactic acid and butyric acid.

## Conclusion

Dietary supplementation of 200 mg/kg EM was beneficial to growth performance and intestinal health of broilers. Important biomarkers such as increased serum and ileal antioxidant capacity, altered microbial communities in the jejunal and ileum, increased fecal SCFAs and lactic acid concentrations, and upregulated ileal-barrier-related gene expression were observed. At the same time, these results revealed the dominant role of *Lactobacillus* in the jejunal and ileal microbiota, which might improve concentrations of lactic acid and SCFAs. Microbial metabolites including lactic and SCFAs can exert beneficial effects on intestinal health and promote the interaction between the intestines and microbiota.

## Data Availability

The data analyzed during the current study are available from the corresponding author on reasonable request.

## Abbreviations

ADFI: Average daily feed intake
ADG: Average daily gain
BW: Body weight
CAT: Catalase
CTC: Chlortetracycline
DAO: Diamine oxidase
EM: Epimedium
F/G: Feed to gain ratio. GSH-Px:Glutathione peroxidase
LDA: Linear discriminant analysis
MDA: Malondialdehyde
NADPH: Nicotinamide adenine dinucleotide phosphate oxidase
NC: Normal control
OTUs: Operational taxonomic units
PLS-DA: Partial least squares discriminant analysis
SCFAs: Short-chain fatty acids
T-AOC: Total antioxidant capacity
